# Chemerin Stimulates Vascular Smooth Muscle Cell Proliferation and Carotid Neointimal Hyperplasia by Activating Mitogen-Activated Protein Kinase Signaling

**DOI:** 10.1371/journal.pone.0165305

**Published:** 2016-10-28

**Authors:** Wei Xiong, Yu Luo, Lin Wu, Feng Liu, Huadong Liu, Jianghua Li, Bihong Liao, Shaohong Dong

**Affiliations:** 1 Department of Cardiology, The Second Clinical Medical College of Jinan University, Shenzhen People’s Hospital, NO. 1017, Dongmen North Road, Shenzhen, 518020, China; 2 Department of Cardiology, Peking University First Hospital, No. 8, Xishiku Street, Beijing, 100034, China; Albany Medical College, UNITED STATES

## Abstract

Vascular neointimal hyperplasia and remodeling arising from local inflammation are characteristic pathogeneses of proliferative cardiovascular diseases, such as atherosclerosis and post angioplasty restenosis. The molecular mechanisms behind these pathological processes have not been fully determined. The adipokine chemerin is associated with obesity, metabolism, and control of inflammation. Recently, chemerin has gained increased attention as it was found to play a critical role in the development of cardiovascular diseases. In this study, we investigated the effects of chemerin on the regulation of vascular smooth muscle cells and carotid neointimal formation after angioplasty. We found that circulating chemerin levels increased after carotid balloon injury, and that knockdown of chemerin significantly inhibited the proliferative aspects of vascular smooth muscle cells induced by platelet-derived growth factor-BB and pro-inflammatory chemokines *in vitro* as well as prohibited carotid neointimal hyperplasia and pro-inflammatory chemokines *in vivo* after angioplasty. Additionally, inhibition of chemerin down-regulated the expression of several proteins, including phosphorylated p38 mitogen-activated protein kinase, phosphorylated extracellular signal regulated kinase 1/2, nuclear factor-kappa B p65, and proliferation cell nuclear antigen. The novel finding of this study is that chemerin stimulated vascular smooth muscle cells proliferation and carotid intimal hyperplasia through activation of the mitogen-activated protein kinase signaling pathway, which may lead to vascular inflammation and remodeling, and is relevant to proliferative cardiovascular diseases.

## Introduction

In the past decades, percutaneous coronary intervention has become the most critical revascularization procedure for coronary artery disease, however, restenosis after angioplasty is a major problem associated with this procedure [1]. Although the drug-eluting stent has been confirmed to effectively reduce the rate of in-stent restenosis and target lesion revascularization [2], prolonged anti-platelet agents are required to prevent the risk for late thrombosis [3]. As yet, it remains unknown whether the new biodegradable stent will provide an effective solution [4].

Clarifying the pathogeneses of proliferative cardiovascular diseases such as hypertension, atherosclerosis, and restenosis after angioplasty will contribute to the development of preventive measures and therapies. Inflammation and the immune response play crucial roles in the pathophysiological processes of these diseases [5]. Finding novel targets to treat the inflammatory and immune processes will be of great clinical benefit.

Discovered in 1997, chemerin was originally associated with the normal physiology of human skin [6]. Chemerin was identified as a chemoattractant that stimulates the recruitment of dendritic cells and macrophages to lymphoid organs and the sites of injury [7]. It was demonstrated that chemerin, acting through its major receptor, chemokine-like receptor 1 (CMKLR1), potently increased the production of pro-inflammatory cytokines such as tumor necrosis factor-α (TNF-α), interleukin (IL)-1, and IL-6 as well as matrix metallopeptidase (MMP)-9 [8].

Data from research on obesity, diabetes, and metabolic syndrome suggest that chemerin is an adipokine [9–11]. Yet, novel roles for chemerin in cardiovascular diseases have recently been revealed. In patients with coronary artery disease, serum chemerin levels significantly increased and positively correlated with systolic blood pressure [12]. Chemerin is also reported to be associated with endothelial activation and atherosclerosis in rheumatoid arthritis patients [13]. In patients with dilated cardiomyopathy, plasma concentrations of chemerin were significantly elevated and correlated with the N-terminal pro-brain natriuretic peptide level but negatively correlated with left ventricular ejection fraction [14]. Chemerin, acting through CMKLR1, was also reported to significantly induce endothelial angiogenesis as well as arterial contraction [8, 15]. These findings suggest that chemerin may play a critical role in the development of proliferative cardiovascular diseases.

In this study, we examined the possible roles of chemerin in the proliferative aspects of vascular smooth muscle cells (VSMCs) of mice *in vitro* and in a rat model of neointimal hyperplasia after angioplasty *in vivo* to gain a better understanding of its role in cardiovascular diseases.

## Materials and Methods

### Ethics statement

This study was carried out in strict accordance with the recommendations in the Guide for the Care and Use of Laboratory Animals of the National Institutes of Health. The protocol was approved by the Animal Ethics Committee of Shenzhen People’s Hospital. All efforts were made to minimize suffering.

### Construction of lentiviral vector

Three pairs of short hairpin RNA (shRNA) sequences targeted to knockdown the coding regions of mouse and rat chemerin mRNA were designed and synthesized. The best shRNA was chosen following real-time PCR analysis. The sequences were: sense, ATTCCGACACCCAAGGATATGTCTTTCAAGAGAAGACATATCCTTGGGTGTCTTTTTT; and anti-sense, AATTAAAAAAGACACCCAAGGATATGTCTTCTCTTGAAAGACATATCCTTGGGTGTCG. This shRNA was cloned into the pLVX-shRNA vector to produce the recombinant pLVX-chemerin-shRNA vector. After sequencing, *Escherichia coli* containing the pLVX- chemerin-shRNA vector was cultured and the vector was extracted.

Recombinant lentivirus was produced according to the protocol provided in the Lenti-X Lentiviral Expression Systems kit (Clontech Laboratories, Mountain View, CA). The pLVX-chemerin-shRNA vector and assistant vectors were co-transduced into 293T cells (ATCC, Manassas, VA). After 48-h culture, packaged lentivirus in the supernatant was collected and purified using ultracentrifugation. Mouse VSMCs were cultured and subsequently infected with the recombinant lentivirus. Chemerin mRNA and protein in VSMCs infected with the lentivirus were verified using real-time PCR and Western blotting.

### VSMCs culture

Primary VSMCs were isolated and cultured according to previously described method [16]. Briefly, male NIH mice (50–100 g) were killed by cervical dislocation. Thoracic aortic medias were obtained and propagated in high-glucose Dulbecco’s modified Eagle’s medium (Gibco, Thermo Fisher Scientific, Waltham, MA) with 10% heat-inactivated fetal bovine serum (Gibco) in a humidified incubator containing 5% CO_2_ at 37°C. Immunofluorescence staining of α-smooth muscle actin was performed to verify the purity of VSMCs.

### Carotid artery balloon injury model

Adult male Sprague-Dawley rats (280 ± 20 g, n = 80) were housed in specific pathogen-free animal rooms on 12-h light/dark cycle at 22 ± 2°C and 40–70% humidity with access to food and water *ad libitum*. The carotid artery balloon injury model was constructed as previously described [17]. Chloral hydrate (0.3 g/kg) was intraperitoneally injected to anaesthetize rats before the operation. Briefly, left common carotid arteries were injured using 2 mm × 20 mm balloon catheters (Medtronic, Dublin, Ireland). The balloon was inflated to 151.99 kPa (1.5 atm) for 30 s before being drawn back and subsequently advanced. The procedure was repeated three times. Sham rats underwent the same protocol except for induction of the balloon injury.

After angioplasty, the chemerin gene knockdown lentivirus (CheKo) and the chemerin gene control lentivirus (Vector) (1 × 10^8^ plaque forming units/mL, 50 μL) were infused into the common carotid arteries and reserved for 30 min. At 72 h after the operation, left common carotid arteries, which had undergone balloon injury, were isolated and real-time PCR was performed to detect local chemerin mRNA. The right common carotid arteries, which had not undergone balloon injury, were isolated and used as controls.

### Chemerin expression in carotid artery

To explore the source of chemerin, common carotid arteries were isolated at day 7 after angioplasty. Immunohistochemistry analysis was performed using chemerin antibody (Abcam, Cambridge, UK). The percentage of chemerin-positive VSMCs to all cells in carotid artery tissues was calculated in microscope (Olympus, Tokyo, Japan).

### VSMCs proliferation in vitro

VSMCs were cultured for 72 h and growth curves were drawn. Platelet-derived growth factor-BB (PDGF-BB, 20 ng/mL, Sigma-Aldrich, St. Louis, MO) was used to induce VSMCs proliferation. Proliferation properties were investigated using the bromodeoxyuridine (BrdU, Sigma-Aldrich) incorporation assay after VSMCs had been cultured for 48 h.

### VSMCs proliferation in vivo

Common carotid arteries were isolated at day 7 after angioplasty. BrdU (100 mg/kg) was intraperitoneally injected at 24 h before isolation. BrdU incorporated into the intima of the common carotid artery was investigated using immunohistochemistry, and the percentage of positive VSMCs was calculated in microscope (Olympus).

### Morphometric analysis of carotid artery

Common carotid arteries were isolated at day 14 after the operation. Samples were embedded and stained with hematoxylin and eosin. Morphometric analysis of the carotid artery was performed in microscope (Olympus). Neointima and media areas were identified and the ratio of neointimal area to media area was calculated.

### Enzyme-linked immunosorbent assay

An enzyme-linked immunosorbent assay (ELISA) kit was used to examine serum chemerin levels before angioplasty and at day 3, 7, and 14 post angioplasty, according to the manufacturer‘s protocol. Supernatant in cultured VSMCs and serum from rats were collected to detect IL-1β, IL-6, TNF-α, and monocyte chemotactic protein-1 (MCP-1) levels using ELISA kits after VSMCs had been cultured for 48 h or at day 7 post angioplasty.

### Western blotting

Proteins were isolated from VSMCs after cells had been cultured for 72 h and from common carotid arteries at day 14 after the operation. Protein levels of chemerin (Abcam), phosphorylated p38 mitogen-activated protein kinase (*p*-p38-MAPK, Santa Cruz Biotechnology, Santa Cruz, CA), phosphorylated extracellular signal regulated kinase 1/2 (*p*-ERK 1/2, Santa Cruz Biotechnology), phosphorylated c-Jun N-terminal kinase (*p*-JNK, Cell Signaling Technology, Danvers, MA), nuclear factor-kappaB p65 (NF-κBp65, Cell Signaling Technology), and proliferating cell nuclear antigen (PCNA, Novus Biologicals, Littleton, CO) were investigated using Western blotting analysis.

### Statistical analysis

Statistical analyses were performed using SPSS 12.0 (SPSS Inc., Chicago, IL). Data are mean ± standard error. For relative gene expression, mean value of the Vehicle group was defined as 100%. Differences were compared using ANOVA. A *p-*value <0.05 was considered statistically significantly. All experiments were performed at least triplicate.

## Results

### Serum chemerin was elevated by carotid angioplasty

**Fig 1:**
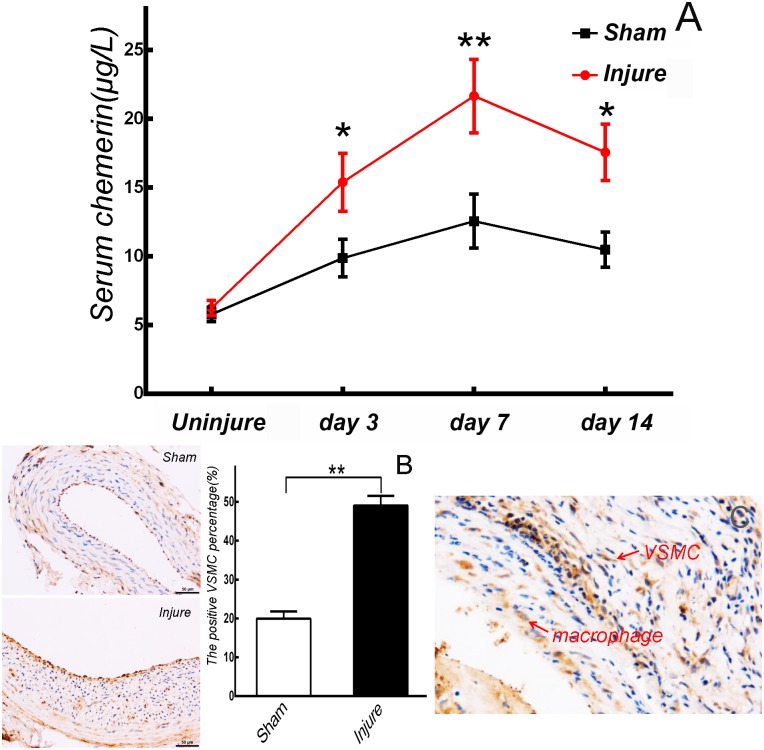
Serum chemerin was significantly elevated by carotid angioplasty. (A) Compared with Sham rats (n = 6), serum chemerin significantly increased in carotid-injured rats (n = 6) from day 3 to day 14 post-angioplasty. * *p*<0.05, ** *p*<0.01. (B, C) Chemerin was primarily produced in VSMCs and endothelial cells after carotid angioplasty. ** *p*<0.01, n = 6.

Carotid neointimal hyperplasia was induced by angioplasty. The serum chemerin levels between Sham and carotid-injured rats (Injure) showed no significant differences before angioplasty (*p>*0.05). After carotid angioplasty, serum chemerin levels significantly increased from day 3 (*p*<0.05), peaked at day 7 (*p*<0.01), and were maintained until day 14 (*p*<0.05) compared with Sham rats (Fig 1A).

Immunohistochemistry analysis indicated that chemerin was primarily located in the cytoplasm of VSMCs and endothelial cells after carotid angioplasty, and was rarely located in macrophages. Compared with Sham rats, the percentage of chemerin-positive VMSCs in rats that underwent carotid angioplasty significantly increased (*vs*. Sham, *p*<0.01. Fig 1B and 1C).

### Knockdown of chemerin inhibited VSMCs proliferation in vitro

**Fig 2:**
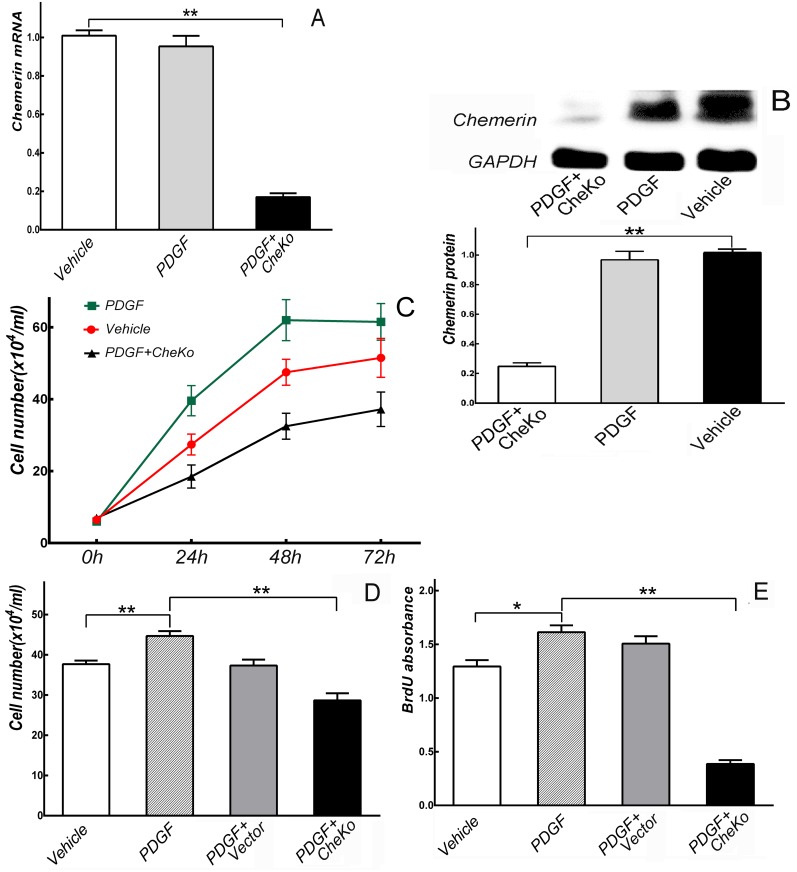
Knockdown of chemerin inhibited VSMCs proliferation in *vitro*. (A, B) Chemerin mRNA and protein levels in VSMCs transfected with lentivirus significantly decreased. ** *p*<0.01, n = 3. (C) Growth curve of VSMCs. (D, E) VSMCs proliferation induced by PDGF-BB (20ng/mL) were significantly inhibited by knockdown of chemerin at 48 h. * *p*<0.05, ** *p*<0.01, n = 6.

The mRNA and protein levels of chemerin were determined using real-time PCR and Western blotting respectively. Compared with normal VSMCs (Vehicle) and PDGF-treated VSMCs (PDGF), chemerin mRNA and protein levels in lentivirus-infected VSMCs (PDGF+CheKo) significantly decreased (*p*<0.01) (Fig 2A and 2B).

After VSMCs were incubated for 48 h, proliferation properties were investigated using cell counting and BrdU analysis. As shown in Fig 2C–2E, PDGF-BB (20 ng/mL) notably induced VSMCs proliferation at 48 h (*vs*. Vehicle, *p*<0.05). In lentivirus vector VSMCs (PDGF+vector) and PDGF+CheKo VSMCs, PDGF-BB (20ng/mL) was both rendered to induce cell proliferation. However, the VSMCs proliferation induced by PDGF-BB were significantly inhibited by the depletion of chemerin at 48 h (*vs*. PDGF, *p*<0.05). In contrast, cells proliferation did not show significant differences between PDGF+Vector VSMCs and PDGF VSMCs (*p>*0.05).

### Knockdown of chemerin suppressed carotid intimal hyperplasia in vivo

**Fig 3:**
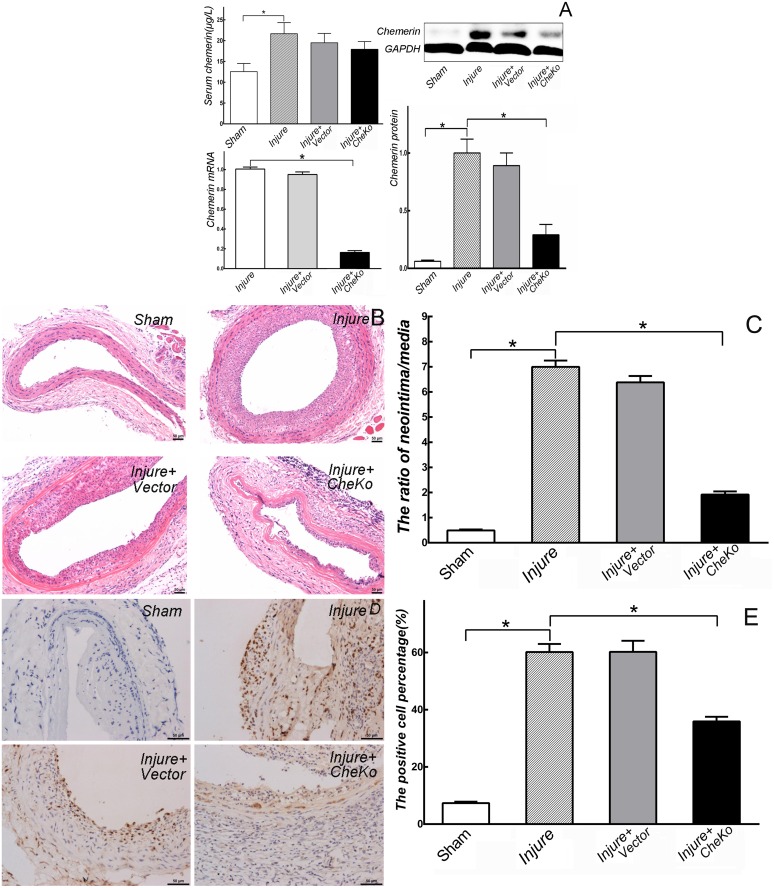
Knockdown of chemerin suppressed intimal hyperplasia of carotid arteries in *vivo*. (A) The increased mRNA and protein levels of chemerin induced by angioplasty in carotid arteries were significantly reduced by local perfusion of lentivirus (n = 3), while serum chemerin levels showed no significant changes after lentivirus perfusion (n = 6). * *p*<0.01. (B, C) Neointimal hyperplasia of carotid induced by angioplasty and the increased ratio of neointimal area to medial area were inhibited by knockdown of chemerin at day 14 post- angioplasty. * *p*<0.01, n = 6. (D, E) VSMCs proliferation in carotid arteries induced by angioplasty and the increased BrdU-positive VSMCs were inhibited by knockdown of chemerin at day 14 post-angioplasty. * *p*<0.01, n = 6.

Chemerin mRNA and protein levels in carotid arteries were significantly decreased by local perfusion of lentivirus (Injure+CheKo *vs*. Injure, *p*<0.01. Fig 3A). However, this decrease caused by lentivirus did not arise in serum chemerin (Injure+CheKo *vs*. Injure, *p*>0.05. Fig 3A).

Compared with uninjured carotid arteries (Sham), injured carotid arteries that underwent angioplasty (Injure) exhibited remarkable neointimal hyperplasia (Fig 3B). The ratio of neointima/media in injured rats significantly increased (*vs*. Sham, *p*<0.01). When chemerin of carotid artery was knocked down (Injure+CheKo), the neointimal hyperplasia induced by angioplasty was significantly suppressed (*vs*. Injure, *p*<0.01. Fig 3B and 3C).

BrdU incorporation analysis was performed to investigate VSMCs proliferation *in vivo*. Compared with Sham rats, the percentage of BrdU-positive VSMCs in injured rats notably increased (*p*<0.01. Fig 3D). When chemerin was knocked down in injured rats, the percentage of BrdU-positive VSMCs in carotid arteries simultaneously reduced (*vs*. Injure, *p*<0.01. Fig 3D and 3E).

### Knockdown of chemerin inhibited up-regulation of pro-inflammatory cytokines

**Fig 4:**
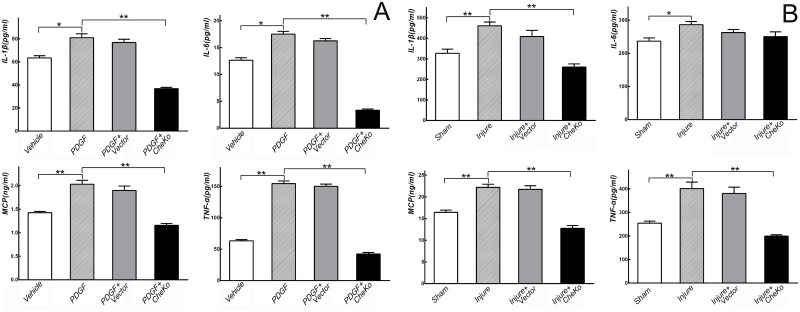
Knockdown of chemerin inhibited activation of pro-inflammatory factors. (A) Elevated pro-inflammatory factors induced by PDGF-BB (20ng/mL) in cultured VSMCs at 48 h were reduced by knockdown of chemerin. * *p*<0.05, ** *p*<0.01, n = 6. (B) Elevated serum pro-inflammatory factors induced by carotid angioplasty in rats at day 7 post-angioplasty were decreased by knockdown of chemerin in carotid tissues, except for IL-6. * *p*<0.05, ** *p*<0.01, n = 6.

To evaluate the relationship between chemerin and inflammation, ELISA was used to determine levels of IL-1β, IL-6, TNF-α, and MCP-1 in cultured VSMCs *in vitr*o and carotid arteries *in vivo*. Compared with Vehicle, PDGF-BB (20 ng/mL) increased IL-1β, IL-6, TNF-α, and MCP-1 levels in VSMCs at 48 h (*p*<0.05). Up-regulation of these pro-inflammatory factors were significantly inhibited by knockdown of chemerin in cultured VSMCs (*vs*. PDGF, *p*<0.05. Fig 4A). Serum levels of IL-1β, IL-6, TNF-α, and MCP-1 in carotid-injured rats significantly increased at day 7 post angioplasty (*vs*. Sham, *p*<0.05). When chemerin of carotid arteries was knocked down in injured rats, serum levels of IL-1β, TNF-α, and MCP-1 were simultaneously suppressed (*vs*. Injure, *p*<0.05. Fig 4B). Interestingly, the serum level of IL-6 did not significantly decrease in Injure+CheKo rats (*p>*0.05).

### Knockdown of chemerin decreased proteins associated with MAPK

**Fig 5:**
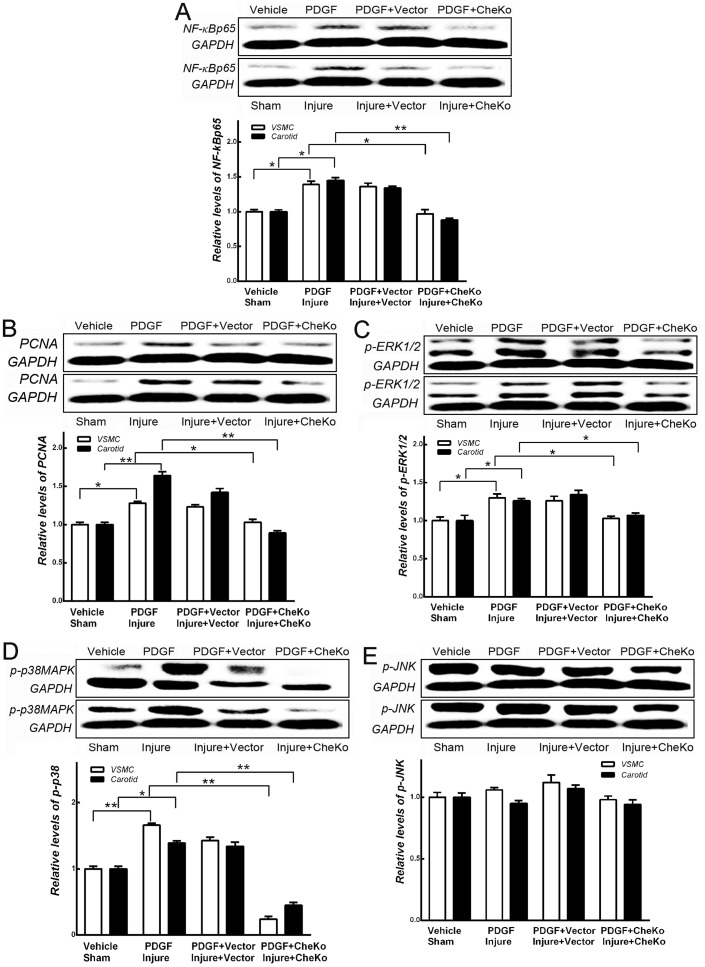
Knockdown of chemerin decreased proteins associated with p-p38-MAPK. Protein levels of NF-κBp65 (A), PCNA (B), *p*-ERK 1/2 (C), and *p*-p38-MAPK (D) in VSMCs and carotid tissues were up-regulated by PDGF-BB (20ng/mL) or carotid angioplasty. Up-regulation of these factors was significantly inhibited by knockdown of chemerin in cultured VSMCs or in injured carotid tissues. The protein levels of *p*-JNK showed no significant changes. * *p*<0.05, ** *p*<0.01, n = 5.

To identify the potential molecular mechanism by which chemerin regulates VSMCs proliferation and carotid neointimal hyperplasia, protein levels of NF-κBp65, PCNA, *p*-ERK 1/2, *p*-p38-MAPK, and *p*-JNK were examined using Western blotting. Protein levels of NF-κBp65 (Fig 5A), PCNA (Fig 5B), *p*-ERK 1/2 (Fig 5C), and *p*-p38-MAPK (Fig 5D) in VSMCs and local carotid tissues were found to be up-regulated by PDGF-BB (20 ng/mL) (*vs*. Vehicle, *p*<0.05) or carotid angioplasty (*vs*. Sham, *p*<0.05). However, up-regulation of these proteins was significantly inhibited when chemerin was knocked down in cultured VSMCs (*vs*. PDGF, *p*<0.05) or injured carotid tissues (*vs*. Injure, *p*<0.05). Protein levels of *p*-JNK in cultured VSMCs and carotid tissues showed no significant differences when chemerin was knocked down.

## Discussion

In the present study, we found that chemerin, which has previously been associated with obesity and metabolic syndrome, is capable of promoting VSMCs proliferation *in vitro* and carotid neointimal formation *in vivo*. A novel finding of this study is that chemerin may act as a potential biomarker for the diagnosis of atherosclerosis and restenosis after percutaneous coronary intervention as well as a prospective target for therapy of the diseases.

Chemerin, usually defined as an adipokine, is predominantly produced in liver and white adipose tissue [9, 18]. Chemerin plays a crucial role in adipogenesis and development [18–20]. Evidences of chemerin and one of its receptors, CMKLR1, being present in β-islet cells raised a possible correlation between chemerin and diabetes mellitus [11, 21–22]. The finding that chemerin is associated with tumor progression and outcome suggested a growth factor role for chemerin [23–25]. In squamous cell carcinoma of the tongue, elevated chemerin significantly correlated with poor differentiation and poor clinical outcome, indicating its role as a novel prognostic factor and a new target for therapy in tumors [25]. Increased level of chemerin could induce the expression of MMP-2, MMP-9, MMP-7, and vascular endothelial growth factor, suggesting that chemerin is associated with the growth and remodeling processes of blood vessels [8, 15]. Chemerin promoted contraction of the rat thoracic aorta, suggesting its role as an endogenous vasoconstrictor [15]. Most recently, the effect of chemerin as a growth factor in vascular biology was investigated. It was reported that chemerin significantly induced rat arterial smooth muscle cells proliferation as well as migration and increased systolic blood pressure [26]. It was also reported that elevated circulatory and local (periaortic and epicardial adipose tissue) chemerin levels significantly increased and positively correlated with the severity of coronary artery disease [12, 27–29].

In the current study, we discovered for the first time that knockdown of chemerin attenuated aortic smooth muscle cell proliferation induced by PDGF-BB as well as intimal hyperplasia of the carotid artery induced by angioplasty. It has previously been reported that chemerin induced growth activity of human endothelial cells by activating PI3K/Akt and MAPK pathways in a dose-dependent manner [8]. Chemerin stimulated the accumulation of natural killer cells from pregnant women by up-regulating ERK expression [30]. Another study reported that chemerin increased mouse myoblasts proliferation and suppressed differentiation through the ERK 1/2 signaling pathway [31]. Our findings, which are consistent with previous reports, supported the possibility that chemerin stimulates VSMCs proliferation and intimal hyperplasia of carotid artery by activating the MAPK signaling pathway.

Foam cells and VSMCs play substantial roles in atheromatous plaque formation and progression. It is established that significantly elevated expression of chemerin in perivascular adipocytes, VSMCs, and foam cells is positively associated with vascular inflammation and coronary atherosclerosis severity [27–28]. Acting through CMKLR1, chemerin can recruit and activate macrophages/foam cells to aggravate vascular inflammation and contribute to atherosclerotic lesion [32–33]. Evidence also demonstrated that chemerin modulated VSMCs phenotype and correlated with atherosclerosis development [34]. However, little literature revealed that either VSMCs or foam cells is the steady source of chemerin.

In the current study, we found that chemerin was predominantly originated in the cytoplasm of VSMCs and endothelial cells after carotid angioplasty, and was scarcely produced by macrophages. The expression of chemerin in the cytoplasm of VSMCs was significantly elevated by carotid angioplasty. These results suggest that VSMCs and endothelial cells are the origin of chemerin. However, as this was an initial study, further evidences are required to confirm this suggestion.

It had been demonstrated that three receptors bind to chemerin: CMKLR1, chemokine CC motif receptor-like 2, and G protein-coupled receptor 1 [35–37]. Among these receptors, CMKLR1 is responsible for chemerin-associated signal transduction and chemotactic action. CMKLR1 expression has been confirmed in adipocyte, vascular endothelial cell, and VSMC [8, 9, 34]. Interestingly, CMKLR1 is also present in human platelets and significantly promoted ADP-stimulated platelet aggregation via P2Y12, an important platelet receptor [38]. These findings indicate the potential role of CMKLR1 in vascular inflammation and platelet activation caused by pathological cardiovascular events. However, in the current study, the role of CMKLR1 in carotid neointimal hyperplasia was not investigated. Therefore, whether the effect of chemerin stimulating carotid neointimal hyperplasia is mediated by CMKLR1 or by the other two receptors remains unclear.

In conclusion, we for the first time found that chemerin induced aortic smooth muscle cells proliferation and carotid intimal hyperplasia via activation of MAPK signaling, which may lead to vascular inflammation and remodeling. Further research is required to better understand the role of chemerin and its receptors in the pathophysiologic processes of cardiovascular diseases and their possible use as therapeutic targets.
